# Functional Analysis of the Nitrogen Metabolite Repression Regulator Gene *nmrA* in *Aspergillus flavus*

**DOI:** 10.3389/fmicb.2016.01794

**Published:** 2016-11-25

**Authors:** Xiaoyun Han, Mengguang Qiu, Bin Wang, Wen-Bing Yin, Xinyi Nie, Qiuping Qin, Silin Ren, Kunlong Yang, Feng Zhang, Zhenhong Zhuang, Shihua Wang

**Affiliations:** ^1^Key Laboratory of Pathogenic Fungi and Mycotoxins of Fujian Province, Key Laboratory of Biopesticide and Chemical Biology of Education Ministry, School of Life Sciences, Fujian Agriculture and Forestry UniversityFuzhou, China; ^2^State Key Laboratory of Mycology, Institute of Microbiology, Chinese Academy of SciencesBeijing, China

**Keywords:** *Aspergillus flavus*, nitrogen metabolism, *nmrA*, aflatoxins, AreA

## Abstract

In *Aspergillus nidulans*, the nitrogen metabolite repression (NMR) regulator NmrA plays a major role in regulating the activity of the GATA transcription factor AreA during nitrogen metabolism. However, the function of *nmrA* in *A. flavus* has not been previously studied. Here, we report the identification and functional analysis of *nmrA* in *A. flavus*. Our work showed that the amino acid sequences of NmrA are highly conserved among *Aspergillus* species and that *A. flavus* NmrA protein contains a canonical Rossmann fold motif. Deletion of *nmrA* slowed the growth of *A. flavus* but significantly increased conidiation and sclerotia production. Moreover, seed infection experiments indicated that *nmrA* is required for the invasive virulence of *A. flavus*. In addition, the Δ*nmrA* mutant showed increased sensitivity to rapamycin and methyl methanesulfonate, suggesting that *nmrA* could be responsive to target of rapamycin signaling and DNA damage. Furthermore, quantitative real-time reverse transcription polymerase chain reaction analysis suggested that *nmrA* might interact with other nitrogen regulatory and catabolic genes. Our study provides a better understanding of NMR and the nitrogen metabolism network in fungi.

## Introduction

*Aspergillus flavus* is ubiquitous in soil and can infect or contaminate a wide range of organic nutrient sources, such as economically important commodities, insects, animal carcasses, and even immunocompromised humans and animals (Amaike and Keller, 2011; Yu, 2012; Zhang et al., 2014; Bai et al., 2015a,b; Zhang et al., 2015). Aflatoxins (AFs), mainly produced by *A. flavus* and *A. parasiticus*, have been identified as a class of the most toxic and carcinogenic secondary metabolites of fungi. AF contamination of agricultural grains is not only a significant food safety concern but also an economic concern for the agricultural industry worldwide (Yu, 2012; Amaike et al., 2013; Yang et al., 2015). To develop effective and novel strategies against AF contamination, it is a necessity to investigate the molecular mechanisms by which AF biosynthesis is regulated in *A. flavus*.

It has been proposed that nitrogen limitation induces the expression of infection-related genes in plant pathogenic fungi (Snoeijers et al., 2000; López-Berges et al., 2010). Microorganisms can use a great many nitrogen sources, and it has become widely accepted that glutamine is a key effector of nitrogen metabolite repression (NMR), a regulatory circuit in filamentous fungi that ensures the preferential use of superior nitrogen sources such as ammonium and glutamine over a broad array of non-preferred compounds (Caddick et al., 1994; Magasanik and Kaiser, 2002; Wong et al., 2008; Wiemann and Tudzynski, 2013; Tudzynski, 2014). The signaling pathway that orchestrates NMR is complex and refined. The positive-acting GATA family transcription factor AreA functions in NMR to allow utilization of preferred nitrogen sources ammonium and glutamine (Wong et al., 2008; Fernandez et al., 2012; Tudzynski, 2014). However, AreA loss-of-function mutants are unable to use non-preferential nitrogen sources other than ammonium and glutamine (Arst and Cove, 1973; Marzluf, 1997). In the presence of ammonium and glutamine, the nitrogen metabolite repressor NmrA interacts with AreA to prevent nitrogen catabolic gene expression; however, in the presence of less preferred nitrogen sources such as nitrate, NmrA dissociates from AreA, allowing AreA to activate the expression of genes involved in alternative nitrogen source usage (Caddick et al., 1994; Andrianopoulos et al., 1998; Wilson and Arst, 1998; Fernandez et al., 2012; Tudzynski, 2014).

The function of NmrA in nitrogen source metabolism in *A. flavus* has not been well characterized (Andrianopoulos et al., 1998; Schönig et al., 2008; Wagner et al., 2010; Zhao et al., 2010). In this study, we identified and cloned the *nmrA* gene from *A. flavus* and characterized *nmrA* deletion and complementation mutants. We demonstrate that *nmrA* plays a negative role in NMR and nitrogen metabolism. Furthermore, *nmrA* appears to be involved in AF biosynthesis, conidiation, sclerotia formation, invasive virulence, and stress responses.

## Materials and Methods

### Fungal Strains and Growth Conditions

Fungal strains and plasmids used in this study are listed in **Supplementary Table S1**. The *A. flavus* strain PTSΔ*ku70*Δ*pyrG* (Chang et al., 2010), a uracil auxotroph, was purchased from the Fungal Genetics Stock Center (School of Biological Sciences, University of Missouri, Kansas City, MO, USA) and used for gene deletion (Yang et al., 2016). Wild-type (WT) *A. flavus* and the transformants generated in this study were grown on yeast extract–sucrose (YES) agar, yeast extract–glucose agar, or yeast extract–glucose agar with trace elements, uracil, and uridine (Yang et al., 2016). YES medium, potato dextrose agar (PDA, Bai and Shaner, 1996), Czapek agar (CA, Becton Dickinson), and glucose minimal medium (GMM, Shimizu and Keller, 2001) were used for mycelial growth assays, sporulation analysis, and AF analysis. All experiments included four replicate plates and were performed at least three times with similar results, and the error was expressed as the standard deviation.

### Sequence Analysis of NmrA

The NmrA sequence (EED47920) from *A. flavus* was originally identified in the NCBI Protein database using BLAST. To verify the existence and the size of the exon in *nmrA*, we cloned the coding sequence into the pET-28a(+) expression vector and sequenced it. The obtained sequence was compared with that reported in the NCBI database. The protein domains were analyzed using InterPro^1^. A phylogenetic tree based on NmrA sequences from *A. clavatus* (UniProt: A1C544), *A. flavus* (UniProt: B8NR87), *A. fumigatus* (UniProt: Q4WEG4), *A. nidulans* (UniProt: Q5AU62), *A. niger* (G3XN01), *A. oryzae* (GenBank: XP_001822655.2), and *A. terreus* (UniProt: Q0CAL7) was constructed with DNAMAN 6.0 software using the neighbor-joining method.

### Targeted Deletion and Complementation of the *nmrA* Gene

To create an *nmrA* deletion mutant, we constructed an AP-pyrG-BP vector by inserting upstream and downstream flanking sequences of the *nmrA* gene on either side of the *pyrG* gene. The upstream and downstream flanking sequences of *nmrA* were amplified from genomic DNA of *A. flavus* WT with primer pairs *nmrA*-P1/*nmrA*-P2 and *nmrA*-P3/*nmrA*-P4, respectively (**Supplementary Table S2**). The *nmrA* deletion mutant (Δ*nmrA*) was complemented with a full-length *nmrA* gene, a 2869 bp fragment, spanning from 1069 bp upstream of the WT *A. flavus nmrA* translation initiation codon to 681 bp downstream of the translation termination codon, to confirm that the phenotype of the Δ*nmrA* mutant was due to deletion of the *nmrA* gene. The full-length *nmrA* gene was amplified from genomic DNA of *A. flavus* WT using the primer pair *nmrA*-CF/*nmrA*-CR (**Supplementary Table S2**), and it contained a constitutive promoter, which controlled *nmrA* gene expression. The complementation plasmid (pPTR I-*nmrA*) was constructed using the backbone of the chromosomal integrating shuttle vector pPTR I DNA (Takara, Japan, Kubodera et al., 2000), which contained the same restriction enzyme recognition sites as the full-length *nmrA* gene and could integrate into *A. flavus* genome randomly. Six out of 15 pyrithiamine resistance transformants were selected for their wild-type growth phenotype in the presence of GMM supplementing ammonium as the sole nitrogen source. We concluded that these transformants had integrated an intact copy of the *A. flavus nmrA* gene into the genome after the *A. flavus nmrA* gene in this plasmid was sequenced to ensure flawlessness of the sequence.

### Mycelial Growth and Stress Assays

Mycelial growth assays were performed on YES medium, PDA, and GMM supplemented with 50 mM glutamine, ammonium, proline, alanine, or sodium nitrite (NaNO_2_). For the stress assays, rapamycin, methyl methanesulfonate (MMS), NaCl, and sorbitol were added to YES medium at the concentrations indicated in the figure legends (Yang et al., 2016). Each plate was inoculated with 1 μL of conidial suspension (4 × 10^4^ conidia/mL) and incubated at 28°C for 4–7 days in the dark. The assays were carried out in triplicate and were repeated three times.

### Observation and Counting of Conidia

Glucose minimal medium agar and PDA were point-inoculated with 1 μL of conidial suspension (4 × 10^4^ conidia/mL) and incubated at 28°C for 5 days in the dark. Three plugs were removed from each plate and the conidia produced by *A. flavus* were suspended in a solution of 0.05% Tween 20 and 7% dimethylsulfoxide (DMSO), and counted in a hemocytometer. Conidial suspensions were viewed with an inverted microscope (Leica Microsystems, Germany).

### Analysis of AF Production

For analysis of AF production, 1 mL of spore suspension (1 × 10^6^ spores/mL) was inoculated in YES, PDA or GMM supplemented with 50 mM glutamine, ammonium, proline, alanine, or sodium nitrite and incubated in the dark at 28°C for 7 days. AFs were extracted from 500 μL of culture filtrate with an equal volume of chloroform. The chloroform layer was transferred to a new 1.5 mL tube and evaporated to dryness at 70°C. Thin-layer chromatography was used to identify AFs. A solvent system consisting of acetone and chloroform (1:9, v/v) was used, and the plates were observed under UV light at 365 nm. For quantitative analysis of AF production, GeneTools image analysis software was used.

### Sclerotia Assays

Sclerotia formation was measured as previously described (Amaike and Keller, 2011). Briefly, GMM supplemented with the indicated nitrogen sources, 2% agar, and 2% sorbitol was overlaid with 1 × 10^4^ spores/plate. Cultures were grown at 37°C in complete darkness for 7–10 days. Plates were sprayed with 70% ethanol to kill and wash away conidia and exposed sclerotia. The sclerotia were collected from 10 mm cores and counted in triplicate. Experiments were repeated three times.

### Seed Infection

Mature live peanut seeds were used to measure the pathogenicity of the WT, Δ*nmrA*, and complementation (Δ*nmrA*::*nmrA*) strains (Duran et al., 2007; Amaike and Keller, 2011). Plates were cultured in the dark at 28°C for 5 days, and the filter paper was moistened daily. Inoculated peanut cotyledons were processed to count conidia and extract AFs, and the methodology of counting conidia and extracting AFs was the same as above described.

### Real-Time Quantitative Reverse Transcription Polymerase Chain Reaction (RT-qPCR)

*Aspergillus flavus* mycelia from the WT, Δ*nmrA*, and Δ*nmrA*::*nmrA* strains were harvested after 48 h of growth. RNA was extracted with TRIzol reagent (Biomarker Technologies, Beijing, China), and then a Nano Drop 2000 and Agilent 2100 were used to evaluate the quality of RNA after total RNA extraction and DNase I treatment. TransScript^®^ All-in-One First-Strand cDNA Synthesis SuperMix was used to synthesize cDNA, and RT-qPCR was performed on a PikoReal^TM^ Real-Time PCR System (Thermo Scientific Inc.) with PikoReal^TM^ 2.2 software using TransStart Top Green qPCR SuperMix (TransGen Biotech, Beijing, China). The RT-qPCR conditions were as follows: 95°C for 7 min and 40 cycles of 95°C for 5 s and 60°C for 30 s. The *A. flavus actin* gene was used as an reference gene to normalize the expression data. The relative expression of genes was calculated using the 2^-ΔΔCt^ method, and standard deviation was calculated from three biological replicates (Livak and Schmittgen, 2001). The gene-specific primers are shown in **Supplementarey Table S3**.

## Results

### Sequence Analysis of NmrA in *Aspergillus*

**FIGURE 1:**
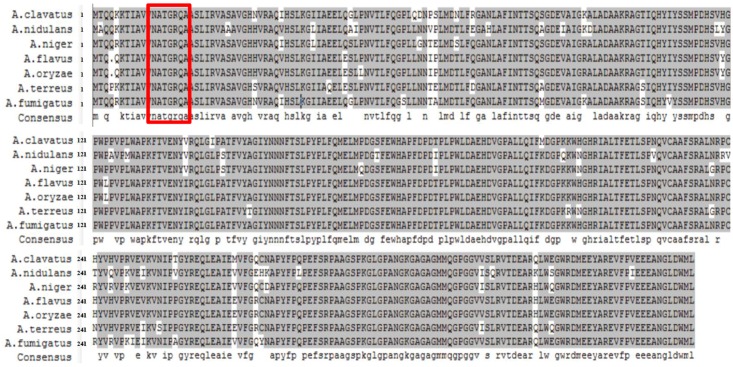
**Sequence alignment of nitrogen metabolite repressor NmrA proteins in *Aspergillus clavatus*, *A. flavus*, *A. fumigatus*, *A. nidulans*, *A. niger*, *A. oryzae*, and *A. terreus*.** DNAMAN 6.0.3.99 software was used for the alignment and presentation. Residues boxed in red mark the Rossmann fold motif.

In *A. nidulans*, NmrA can bind nicotinamide dinucleotides and may have a redox-sensing function, since NmrA is bound with NAD^+^ and NADP^+^ preferentially to NADH and NADPH. In addition, NmrA interacts directly with AreA zinc fingers (Stammers et al., 2001; Kotaka et al., 2008; Zhao et al., 2010). The *A. flavus nmrA* ORF consists of 1,119 bp with one introns, and encodes a putative NMR regulator NmrA with 351 amino acids. *A. flavus* NmrA might function similarly to its ortholog in *A. nidulans*, since the two protein sequences have a similarity of 88.07%. We downloaded the NmrA protein sequences of seven representative *Aspergillus* species (*A. clavatus*, *A. flavus*, *A. fumigatus*, *A. nidulans*, *A. niger*, *A. oryzae*, and *A. terreus*) from FungiDB^2^ and aligned using DNAMAN software version 6.0.3.99 (**Figure 1**). The sequence similarity was as high as 94.81%, demonstrating a highly conserved nature of NmrA in *Aspergillus*. For instance, the NmrA protein sequences in *A. flavus* and *A. oryzae* are only differed at one amino acid between the proteins of, reflecting the evolutionary similarity between them. We also identified a canonical Rossmann fold motif in *A. flavus* NmrA.

### Radial Growth on Various Nitrogen Sources

**FIGURE 2:**
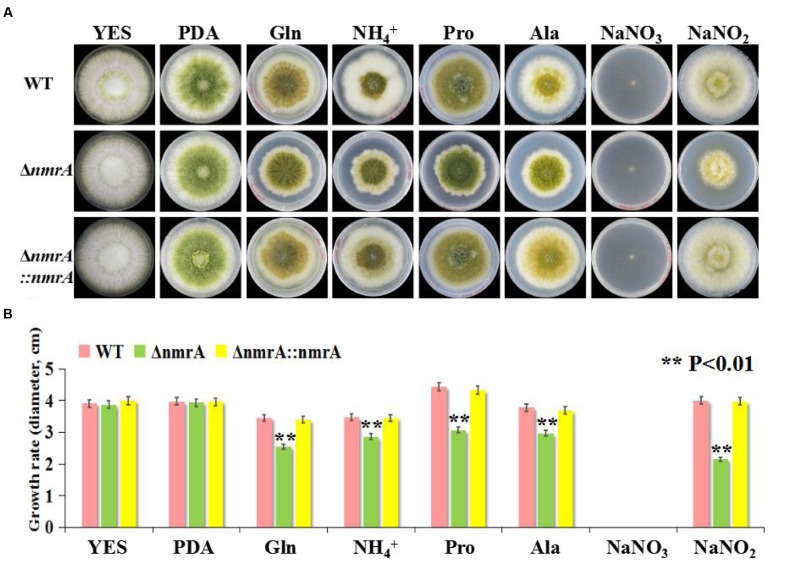
**Utilization of *A. flavus* Δ*nmrA* mutant on different nitrogen sources.** **(A)** Radial growth of mycelia on YES, PDA, and GMM supplemented with 50 mM glutamine, ammonium, proline, alanine, sodium nitrate, and sodium nitrite, respectively, as a sole nitrogen source for 7 days at 28°C in the darkness. The photographs were taken on the 5th day. **(B)** Growth rate of WT, Δ*nmrA* and Δ*nmrA*::*nmrA* strains vegetated on YES, PDA, and GMM supplemented with different nitrogen sources on the 5th day at 28°C in the dark. Asterisk indicated statistical significance at *P* < 0.01.

A targeted gene deletion strategy was employed to determine the roles of *nmrA* in *A. flavus* (**Supplementary Figure S1A**). The *nmrA* gene was successfully replaced with the *pyrG* cassette (**Supplementary Figure S1B**), indicating that the *nmrA* deletion mutant (Δ*nmrA*) was successfully constructed. The Δ*nmrA* strain was complemented by introducing a construct consisting of the *nmrA* open reading frame in the pPTR I vector, which generated the complementary strain Δ*nmrA*::*nmrA*. The Δ*nmrA* and Δ*nmrA*::*nmrA* strains were verified by RT-PCR and RT-qPCR (**Supplementary Figures S1B,C**). The growth rate of the Δ*nmrA* strain consistently matched that of the WT and Δ*nmrA*::*nmrA* strains on YES medium and PDA, but the growth rate was slower than that of the WT and Δ*nmrA*::*nmrA* strains on GMM supplemented with glutamine, ammonium, proline, alanine, or sodium nitrite as the sole nitrogen source (the usual nitrogen source in GMM is sodium nitrate, NaNO_3_) (**Figures 2A,B**). The edges of the mycelial colonies of the Δ*nmrA* strain were more irregular on glutamine, ammonium, and proline than on the other nitrogen sources (**Figure 2A**). It should be mentioned that *A. flavus* strains could not grow normally on sodium nitrate because of a deficiency of the nitrate reductase gene *niaD*. Therefore, sodium nitrite was used in place of sodium nitrate; however, the deletion strain showed retarded growth on this nitrogen source as well. These results suggested that *nmrA* could regulate nitrogen utilization and metabolism.

### Colony Morphology and Conidiation of the Δ*nmrA* Mutant

**FIGURE 3:**
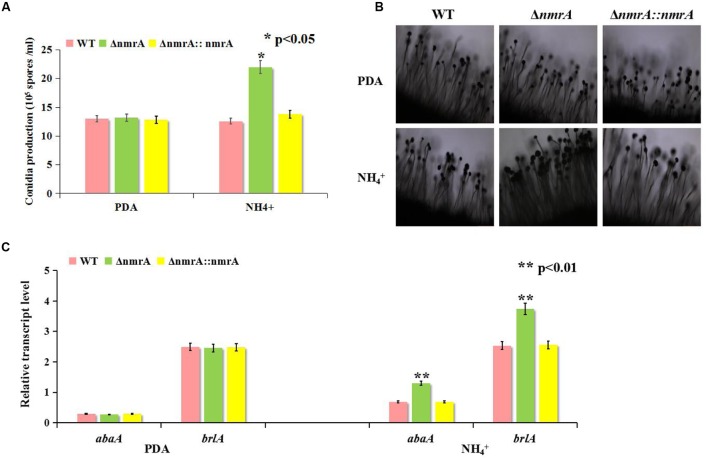
**Deletion of *nmrA* affected conidia production.** Bars represent SE from three independent experiments with three replicates. **(A)** Deletion of *nmrA* resulted in conidiation augment on GMM supplemented with ammonium. Asterisk indicated statistical significance at *P* < 0.05. **(B)** Conidiophores were observed under a light microscope at 12 h after induction with illumination. Scale bar: 200 μm. **(C)** RT-qPCR analysis was performed in the indicated strains germinated on media as described in **(A)**. The asterisks represented a significant difference level of *P* < 0.01.

There was little difference in conidia production among strains when they were germinated on PDA, but the Δ*nmrA* strain produced more spores than the WT and Δ*nmrA*::*nmrA* strains when germination occurred on GMM with ammonium (**Figures 2A** and **3A**). The Δ*nmrA* mutant also produced more conidiophores than the WT and Δ*nmrA*::*nmrA* strains on GMM with ammonium (**Figure 3B**). To gain further insight into the role of NmrA in conidiation, we performed RT-qPCR analysis. The results showed that transcript levels of the conidiation-related genes *abaA* (AFLA_029620) and *brlA* (AFLA_082850) were higher in Δ*nmrA* than in WT *A. flavus* and Δ*nmrA*::*nmrA* when the strains were germinated on GMM with ammonium (Kato et al., 2003; Son et al., 2013), while there was little difference among strains when germination occurred on PDA (**Figure 3C**). Collectively, these results indicated that *nmrA* was likely involved in the regulation of conidiation in *A. flavus*.

### AF Production by the Δ*nmrA* Mutant

**FIGURE 4:**
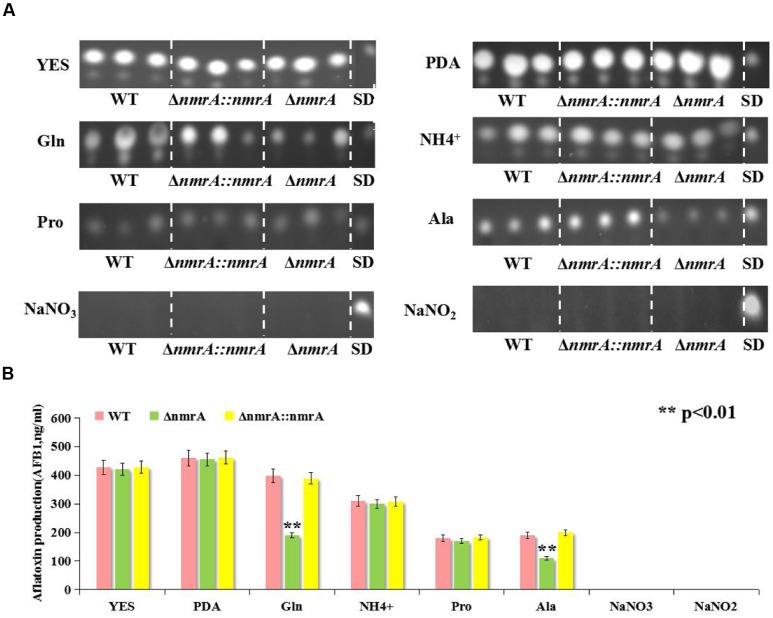
**Aflatoxin (AFB1) assessment of WT, Δ*nmrA* and Δ*nmrA*::*nmrA* strains grown on different culture media.** **(A)** Aflatoxin production of WT, Δ*nmrA* and Δ*nmrA*::*nmrA* strains grown on media as described in **Figure 2**. **(B)** Quantification results of AFB1 of WT, Δ*nmrA* and Δ*nmrA*::*nmrA* strains grown on as **(A)** by Gene Tools analysis system software. SD means standard AFB1. The asterisks represented a significant difference level of *P* < 0.01.

Next, we assessed the production of AFs by the WT, Δ*nmrA*, and Δ*nmrA*::*nmrA* strains on the media described in **Figure 2**. Like the growth rate, AF production was similar among the WT, Δ*nmrA*, and Δ*nmrA*::*nmrA* strains when they were grown on YES or PDA plates (**Figure 4**). However, there were significant decrease in AF production among the strains when they were germinated on GMM supplemented with glutamine and alanine (**Figures 4A,B**). We concluded that *nmrA* might participate in the regulation of AF biosynthesis in *A. flavus*.

### Sclerotia Production by the *nmrA* Deletion Strain

**FIGURE 5:**
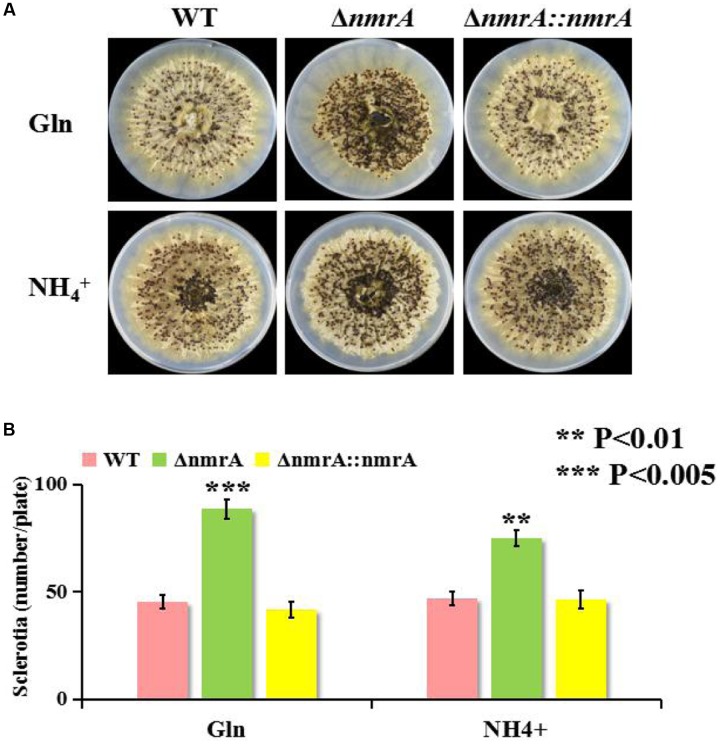
**Sclerotia production of Δ*nmrA* mutant.** **(A)** One representative plate for the WT, Δ*nmrA* and Δ*nmrA*::*nmrA* strains grown on GMM supplemented with 50 mM glutamine and ammonium plus 2% sorbitol to induce sclerotia production at 37°C for 7 days in dark condition. **(B)** Quantity analysis of sclerotia among the strains listed. Asterisks indicated significant differences between each depletion strain relative to the WT and Δ*nmrA*::*nmrA* strains as determined by a Student *T*-test, with ^∗∗^*P* < 0.01, ^∗∗∗^*P* < 0.005.

We found that in addition to suppressing AF biosynthesis, *nmrA* deletion resulted in a concomitant increase in sclerotia production. The Δ*nmrA* strain displayed a more compact and intensive distribution of sclerotia on the plate than the WT and Δ*nmrA*::*nmrA* strains (**Figure 5A**). To some extent, sclerotia production by the Δ*nmrA* mutant was greater on glutamine than on ammonium (**Figure 5B**), indicating that it could be affected by the quality of the nitrogen source. In summary, the effect of the *nmrA* gene on sclerotia production by *A. flavus* was modulated by the nitrogen source.

### Requirement of *nmrA* for Virulence of *A. flavus*

**FIGURE 6:**
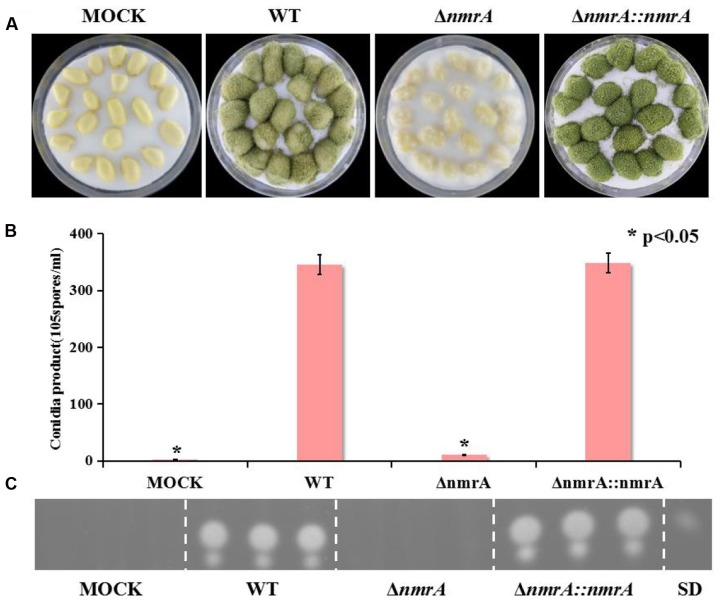
**Pathogenicity assay of Δ*nmrA* mutant.** **(A)** Growth of *A. flavus* WT, Δ*nmrA* and Δ*nmrA*::*nmrA* strains on living peanut cotyledons after 5 days of inoculation. **(B)** Conidia production on peanut cotyledons after inoculation of 5 days. Asterisk indicates statistical significance at *P* < 0.05. **(C)** TLC measurements of aflatoxin (AFB1) extracted from seed in **(A)**. SD means standard AFB1.

Although Wilson examined the role of three NmrA orthologs in *Magnaporthe oryzae* during infection of rice (Wilson et al., 2010), there has been no report on the role of NmrA in infection by *A. flavus.* To dissect the role of NmrA in invasive growth, we assessed the growth of *A. flavus* on peanut seeds. **Figure 6A** shows that the Δ*nmrA* strain was crippled in its ability to colonize and sporulate on host seeds compared with WT *A. flavus* and Δ*nmrA*::*nmrA*. Deletion of *nmrA* also led to statistically significant reductions in conidia production and AF biosynthesis (**Figures 6B,C**), which resulted in the decrease in virulence. These data suggested that *nmrA* deletion reduced the virulence of *A. flavus*.

### Responses of the Δ*nmrA* Mutant to Multiple Stressors

**FIGURE 7:**
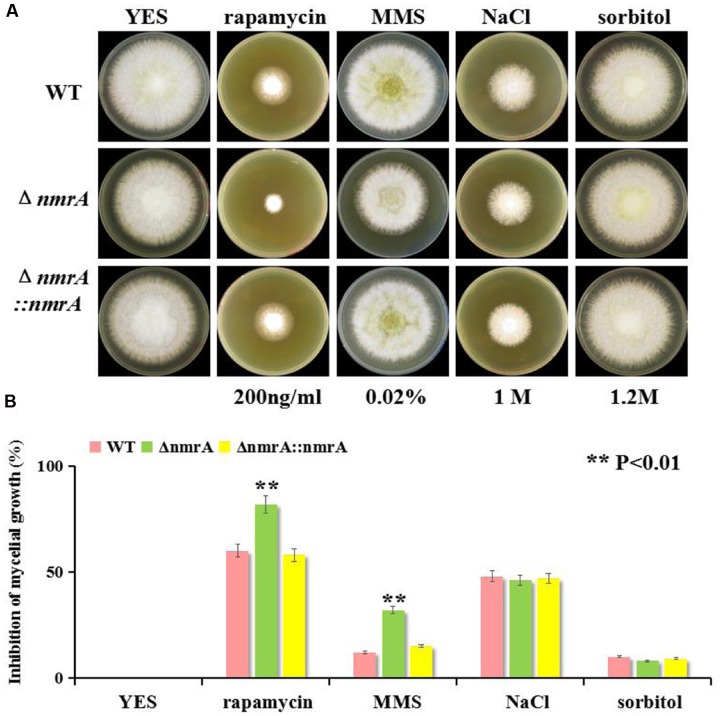
**Sensitivity of the Δ*nmrA* strain to multiple stress reagents.** **(A)** Sensitivity of WT, Δ*nmrA* and Δ*nmrA*::*nmrA* strains to 200 ng/mL rapamycin (specific inhibitor of TOR, Target of Rapamycin), 0.02% MMS (methyl methanesulfonate), 1 M NaCl and 1.2 M sorbitol added in YES agar. **(B)** Inhibition of mycelial growth in the Δ*nmrA* strain to multiple stress reagents. Asterisk indicates statistical significance at *P* < 0.01.

To explore the potential roles of NmrA in responses to environmental stressors, we tested fungal sensitivity to the target of rapamycin (TOR) inhibitor rapamycin, the alkylating agent MMS, and the osmotic stressors NaCl and sorbitol. Previous research has shown that TOR-mediated repression of nitrogen catabolic genes and virulence occurs through MeaB-dependent and MeaB-independent mechanisms in *Fusarium oxysporum* (López-Berges et al., 2010), so it seemed possible that an interaction between NmrA and TOR might be involved in nitrogen metabolism regulation in *A. flavus*. The assay results showed that the Δ*nmrA* mutant was more sensitive to rapamycin and MMS than WT *A. flavus* or Δ*nmrA*::*nmrA*. However, the Δ*nmrA* mutant was not more sensitive to the osmotic stressors (**Figures 7A,B**). Overall, these results indicated that NmrA is associated with the TOR pathway.

### Possible Crosstalk between NmrA and Other Nitrogen Regulatory Genes

**FIGURE 8:**
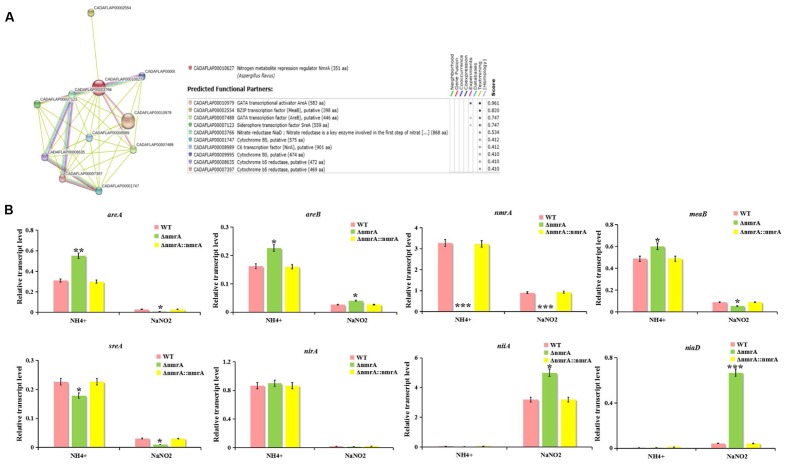
**Effect of nitrogen source and Δ*nmrA* on transcript levels of nitrogen regulatory and catabolic genes.** **(A)** Interaction network analyzed by SMART website. **(B)** RT-qPCR analysis was performed in the indicated strains germinated 30 h on GMM supplemented with 50 mM ammonium or NaNO2 as the sole nitrogen source. Transcript levels of nitrogen regulatory genes *areA*, *areB*, *nmrA*, *meaB*, *sreA*, and *nirA* as well as nitrogen catabolic genes *niaD* and *niiA* in Δ*nmrA* strain (light-green columns) were expressed compared to transcript levels of the WT (pink columns), and Δ*nmrA*::*nmrA* strains (yellow columns). The *A. flavus actin* gene was used as an internal control to normalize the expression data. Bars represent SE from three independent experiments with four replicates each. Asterisks indicated significant differences between each depletion strain relative to the WT and Δ*nmrA*::*nmrA* strains as determined by a Student *T*-test, with ^∗^*P* < 0.05, ^∗∗^*P* < 0.01, ^∗∗∗^*P* < 0.005.

The potential interactive partners of NmrA were identified using the SMART analysis service^3^. The identified partners included the GATA transcriptional activator AreA (AFLA_049870), the bZIP transcription factor MeaB (AFLA_031790), the GATA transcription factor AreB (AFLA_136100), the siderophore transcription factor SreA (AFLA_132440), the nitrate reductase NiaD (AFLA_018810), and the C6 transcription factor NirA (AFLA_093040) (**Figure 8A**). Nitrite reductase NiiA (AFLA_018800) was also analyzed. The possible interactions were analyzed by real-time qRT-PCR. The transcript levels of *areA*, *areB*, *nmrA*, *meaB*, *sreA*, and *nirA* on ammonium were higher than those on nitrite, suggesting that these genes are likely involved in NMR. The finding that the *areA* expression level on ammonium exceeded that on nitrite was unexpected and was probably correlated with nitrate reductase deficiency and the relative richness of the nitrogen sources. However, transcript levels of *niaD* and *niiA* on ammonium were lower than those on nitrite (**Figure 8B**). Furthermore, on ammonium, all transcript levels except that of *sreA* were higher in the Δ*nmrA* strain than in the WT and Δ*nmrA*::*nmrA* strains. In contrast, on nitrite, *areA*, *meaB*, *sreA*, and *nirA* transcript levels were downregulated in the Δ*nmrA* mutant, while *areB*, *niaD*, and *niiA* transcript levels were upregulated, which was consistent with the postulated reverse roles of *areA* and *areB*. Importantly, the finding that the *areA*, *areB*, and *meaB* transcript levels on ammonium were higher in the Δ*nmrA* mutant than in the WT and Δ*nmrA*::*nmrA* strains indicates that the roles of these genes in nitrogen repression might be related to *nmrA.* Moreover, the observation that *niaD* and *niiA* transcript levels on nitrite were significantly higher in the Δ*nmrA* mutant than in WT *A. flavus* and Δ*nmrA*::*nmrA* demonstrates that NmrA strictly repressed transcriptional activation of genes encoding enzymes required for utilization of less favored nitrogen sources. In conclusion, these findings shed light on the complexity and sophistication of the regulatory mechanisms of NMR.

## Discussion

The protein NmrA was defined as a repressor of the GATA transcription factor AreA, which regulates several genes required for utilization of less preferred nitrogen sources (Caddick et al., 1986; Andrianopoulos et al., 1998; Tudzynski, 2014), and the bZIP protein MeaB was proposed to activate NmrA in *A. nidulans* (Wong et al., 2007; López-Berges et al., 2010; Amaike et al., 2013). AreA, MeaB, and NmrA and are conserved in filamentous fungi (Wagner et al., 2010). In this study, we established that NmrA is highly conserved among *Aspergillus* species at the amino acid sequence level, and NmrA of *A. flavus* has a canonical Rossmann fold motif. We then investigated the roles of NmrA in *A. flavus*.

Our work showed that *nmrA* deletion reduced the growth of *A. flavus*, suggesting that *nmrA* participated in nitrogen source metabolism and utilization. Moreover, we also found that there was no difference in mycelial growth among the WT, Δ*nmrA* and Δ*nmrA*::*nmrA* strains grown on Czapek Dox media (carbon source is sucrose), regardless of the nitrogen source (**Supplementary Figure S2**), which was different from the results of GMM (carbon source is glucose), we speculated that NmrA might also participate in carbon catabolite repression (CCR). It has been shown that deletion of *nmr* (an *nmrA* homolog) in *F. graminearum* had little effect on growth or toxin production in the presence of sucrose (Giese et al., 2013). Furthermore, NmrA was suggested to play a role in carbon metabolism (Macios et al., 2012). Therefore, further investigation of the connections of NmrA with nitrogen and carbon metabolism should be carried out. Interestingly, *A. flavus* WT (functional mutations of nitrate reductase) was incapable of growth on culture medium with nitrate instead of nitrite, but *A. flavus* NRRL3357 was incapable of growth on culture medium with nitrite (data not shown). Therefore, we doubted that there was preferential utilization of nitrate over nitrite.

*Aspergillus flavus* differentiates to produce asexual dispersing spores (conidia) or overwintering survival structures called sclerotia (Diener et al., 1987; Horowitz Brown et al., 2008). However, sclerotia are also hypothesized to be degenerate sexual structures and may represent a vestige of cleistothecium production (Geiser et al., 1996; Chang et al., 2001). In this study, *nmrA* deletion resulted in significant increases in conidiation and sclerotia production compared with the WT and Δ*nmrA*::*nmrA* strains. Asexual and sexual processes in filamentous fungi are likely to be oppositely regulated by coordinate mechanisms (Horowitz Brown et al., 2008; Chang et al., 2013; Sarikaya-Bayram et al., 2014), so it was surprising that *nmrA* deletion stimulated both conidiation and sclerotia production. These results indicate that NmrA is likely involved in asexual and sexual development.

Aflatoxins, the most deleterious of natural products, are biosynthesized through an extremely refined and sophisticated pathway. This pathway could be affected by many biotic and abiotic factors, including nutritional factors such as carbon and nitrogen sources and environmental factors such as water activity and temperature (Yu, 2012; Amare and Keller, 2014; Zhang et al., 2014, 2015; Bai et al., 2015a,b). In this study, deletion of *nmrA* resulted in reduced AF production only in the presence of glutamine or alanine, suggesting that the effect of *nmrA* on AF biosynthesis is mediated by nitrogen sources and that additional factors must be involved in nitrogen regulation, particularly in the regulation of AF biosynthesis. Although the influence of *nmrA* deletion on secondary metabolism has not been studied in filamentous fungi, the Δ*nmrA* strain of *F. fujikuroi* did not display differential expression of the gibberellic acid biosynthetic gene (Schönig et al., 2008; Tudzynski, 2014). This was not the case in our study and likely reflected the different expression patterns of Nmr1/NmrA in the regulation of secondary metabolism in *Fusarium* and *Aspergillus*.

In some plant pathogenic fungi, AreA is required for full virulence and contributes to pathogenicity, probably because of the failure of mutants to fully adapt to poor nitrogen conditions during infection (Min et al., 2012; Tudzynski, 2014). Deletion of *nmrA* in this study decreased the virulence of *A. flavus* on peanut seeds, resulting in decreased colonization as reflected by lower conidia production and AF production. This indicates that NmrA could also contribute to virulence and pathogenicity. As far as we know, this is the first report of the role of NmrA in the virulence and pathogenicity of *A. flavus.*

We examined the sensitivity of the Δ*nmrA* strain to several stressors to dissect the role of NmrA in stress responses. The Δ*nmrA* mutant showed increased sensitivity to rapamycin and MMS, indicating that *nmrA* might be responsive to TOR signaling and DNA damage. Besides the TOR cascade, other signaling components seem to be involved in nitrogen sensing and subsequent regulation of secondary metabolism (Tudzynski, 2014), so it was not surprising that NmrA might be responsive to TOR. However, deletion of the *nmr1* gene in *F. fujikuroi* resulted in increased rapamycin resistance (Teichert et al., 2006), which was the opposite of what we observed. Therefore, we suspect that the roles of Nmr1/NmrA in nitrogen regulation are regulated differently by TOR signaling in *Fusarium* and *Aspergillus*. Zhao et al. (2010) reported that NmrA could discriminate between oxidized and reduced dinucleotides and was positioned close to the GATA motif in DNA, so NmrA might play a role in DNA protection.

In *A. nidulans*, *nmrA* transcription is partially regulated by the bZIP transcription factor MeaB (Wong et al., 2007). However, recent studies showed that *nmrA* expression in *A. nidulans* and *F. fujikuroi* is not MeaB-dependent (Wagner et al., 2010; Amaike et al., 2013), and MeaB and AreA mediate nitrogen repression coordinately while also functioning independently (Wong et al., 2007; Wagner et al., 2010). Moreover, the ability of AreA and AreB to respond to carbon status probably depends on NmrA rather than the transcription factor CreA, which mediates CCR in *A. nidulans* (Macios et al., 2012). Similarly, the sugar sensor Tps1 and the inhibitor proteins Nmr1–3 (orthologs of NmrA) are all regulators of CCR in *Magnaporthe oryzae* (Fernandez et al., 2012), again illustrating how carbon and nitrogen metabolism are intimately linked. Furthermore, AreA, AreB, MeaB, and Nmr (a homolog of NmrA) have been implicated in the regulation of secondary metabolite production in *F. fujikuroi* (Andrianopoulos et al., 1998; Mihlan et al., 2003; Schönig et al., 2008; Wagner et al., 2010). Nevertheless, for most nitrogen-regulated secondary metabolites in fungi, the molecular mechanism of the nitrogen dependency is not well understood (Tudzynski, 2014). So we attempted to find links between NmrA and other interactive partners in the study. We searched for possible interactions between *nmrA* and other nitrogen regulatory and catabolic genes using the SMART website. RT-qPCR showed that the expression of these interactive partners was up- or down-regulated in the Δ*nmrA* mutant compared with the WT and Δ*nmrA*::*nmrA* strains in the presence of ammonium and nitrite. However, the detailed interaction networks of NmrA and its interactive partners are still unknown. Therefore, further investigation of the interaction networks of nitrogen regulatory and catabolic genes should be undertaken.

In this study, the *nmrA* gene was identified in *A. flavus*, and characterization of *nmrA* mutants revealed that NmrA plays crucial roles in radial growth, conidia and sclerotia formation, AF production, virulence, and stress responses. *nmrA* may also interact with other nitrogen regulatory and catabolic genes. To our knowledge, this is the first report of the function of NmrA in *A. flavus*. Our study provides new insights into the role of NmrA in regulating nitrogen source metabolism and AF biosynthesis in *A. flavus*.

## Author Contributions

XH, BW, and SW conceived and designed the experiments. XH and W-BY wrote the manuscript. XH and MQ performed the experiments. XH and BW analyzed the data. XN, QQ, SR, KY, FZ, and ZZ contributed reagents/materials/analysis tools. SW supported financially and administratively, final approval of manuscript.

## Conflict of Interest Statement

The authors declare that the research was conducted in the absence of any commercial or financial relationships that could be construed as a potential conflict of interest.

The reviewer FS and handling Editor declared their shared affiliation, and the handling Editor states that the process nevertheless met the standards of a fair and objective review.

## Footnotes

1 http://www.ebi.ac.uk/interpro/

2 http://fungidb.org/fungidb/

3 http://smart.embl-heidelberg.de/
