# Supplementary material for: Functional Analysis of the Nitrogen Metabolite Repression Regulator Gene nmrA in Aspergillus flavus
Source: Front Microbiol. 2016 Nov 25;7:1794. doi: 10.3389/fmicb.2016.01794 (PMC5122588; doi:10.3389/fmicb.2016.01794)
Supplement: Supplementary file 1 [file Presentation_1.pdf]

Supporting Information

Table S1 Fungal strains and plasmids used in this study.

Table S2 Gene-specific primers used for *nmrA* gene knock-out and complementation.

Table S3 Gene-specific primers used for RT-qPCR.

Fig. S1 Schematic representation for *nmrA* deletion and identification of Δ*nmrA* and Δ*nmrA*::*nmrA* strains. (A) Schematic representation of genotyping primers used to verify disruption of the *nmrA* locus replaced by *pyrG* from *A. fumigates* (see Table S1 for the primer sequences). (B) Reverse transcription PCR (RT-PCR) result of WT, Δ*nmrA* and Δ*nmrA*::*nmrA* strains. Gene expression levels were normalized (ΔΔCT analysis) to *β-tubulin*. (C) RT-qPCR results showed that the *nmrA* transcript levels in Δ*nmrA* and Δ*nmrA*::*nmrA* strain were absent and present, respectively, confirming the *nmrA* was successfully knockout in Δ*nmrA* and restored in Δ*nmrA*::*nmrA* (see Table S2 for the primer sequences).

Fig. S2 Radial growth of mycelia on Czapek Dox media supplemented with 50 mM glutamine, ammonium, proline, urea and sodium nitrate, respectively, as a sole nitrogen source on the fifth day at 28°C in the darkness.

Supplemental Tables

Table S1 Fungal strains and plasmids used in this study.

Strains and plasmids name	Related gene type	Source
Aspergillus flavus NRRL3357	Wild type	(Zhang et al., 2015)
PTSΔ*ku70*Δ*pyrG*	Δ*ku70*,Δ*pyrG*	(Chang et al., 2010)
Wild type	Δ*ku70*,Δ*pyrG*::*pyrG*	This study
Δ*nmrA*	Δ*ku70*, Δ*nmrA*::*pyrG*	This study

Δ*nmrA::nmrA*	Δ*ku70*, Δ*nmrA::pyrG*, *ptr-nmrA*	This study
Escherichia coli	*DH5α*	Takara, Japan
pPTR I	pPTR I	Takara, Japan
pPTR I-*nmrA*	pPTR I +*nmrA*	This study

Table S2 Gene-specific primers used for *nmrA* gene knock-out, complementation and NmrA localization.

Primer name	Sequence (5'→3')	Application
PyrG-F	GCCTCAAACAATGCTCTTCACCC	Amplifying selective marker PyrG
PyrG-R	GTCTGAGAGGAGGCACTGATGC	
nmrA-P1	CTGGCGACTTCGTCTGTG	Amplifying upstream AP
nmrA-P2	GGGTGAAGAGCATTGTTTGAGGCCTGGTGGGATCAGGAGCA	
nmrA-P3	GCATCAGTGCCTCCTCTCAGACATCGGTTTCACGGCTCTT	Amplifying downstream BP
nmrA-P4	GCAACGAAGCTCCAAAGT	
nmrA-NF	AGCATTCCGAAGCCCTTAT	Amplifying homologous flagments
nmrA-NR	TCCGTTCCCATCCATCAC	
nmrA-UF	CTGGCGACTTCGTCTGTG	Gene deletion and complementation screen
nmrA-UR	CAGGAGTTCTCGGGTTGTCG	
nmrA-OF	TCCCGCAACCTTTGTCTA	Gene deletion and complementation screen
nmrA-OR	CCTCCAACTGTTCTCGGTAT	

nmrA-CF	ATCAAGCTTCTACCCTTGCCGACT	Amplifying complemented flagment
nmrA-CR	CTAGGTACCCGTAATGTGGGTTTGC	

Table S3 Gene-specific primers used for RT-qPCR. (Notice: the primer pair sequences of *niaD* were not in the mutational site for functional deficiency.)

Gene ID	Primer name	Sequence (5'→3')	Application
AFLA_029620	*abaA/*F	TCTTCGGTTGATGGATGATTTC	RT-qPCR
	*abaA/*R	CCGTTGGGAGGCTGGGT	
AFLA_082850	*brlA/*F	GCCTCCAGCGTCAACCTTC	RT-qPCR
	*brlA/*R	TCTCTTCAAATGCTCTTGCCTC	
AFLA_049870	*areA/*F	CCCCCGTTTCCAACTCGATG	RT-qPCR
	*areA/*R	CGCTGGGTGAGAAGGTGAAT	
AFLA_136100	*areB/*F	TTAGGGGCCGGGTAACGGAA	RT-qPCR
	*areB/*R	TTTCGCCATCGGCTCACCAG	
AFLA_018810	*niaD/*F	CGTGAATGGAGAAGTGTATGAT	RT-qPCR
	*niaD/*R	TCCAGACCAGCAGAGGAGAT	
AFLA_018800	*niiA/*F	AAGTACTACGCAACGCAACAG	RT-qPCR
	*niiA/*R	TTGTGATATGGACAGGATACCC	
AFLA_031790	*meaB/*F	GTCTCCTCGCCGTCTCATGC	RT-qPCR
	*meaB/*R	ATATGCCGAAGCGACACGGG	
AFLA_093040	*nirA/*F	GGAGCAAGCAGCTGGTGTCA	RT-qPCR
	*nirA/*R	TTCATGTCGTGCGAGCCTCA	
AFLA_132440	*sreA/*F	AATGGGTCTTCTGCCTCGCC	RT-qPCR

	*sreA/*R	GGTCCATGGGCTCCGAACTG	
AFLA_005620	*nmrA/*F	ATGGCACGGTCACCGGATTG	RT-qPCR
	*nmrA/*R	GGACGGGAGAATTCGGGCTG	
AFLA_055230	*actin/*F	ACGGTGTCGTCACAAACTGG	RT-qPCR
	*actin/*R	CGGTTGGACTTAGGGTTGATAG	
AFLA_068620	*β-tubulin/F*	TTGAGCCCTACAACGCCACT	RT-qPCR
	β-tubulin/R	TGGTTCAGGTCACCGTAAGAGG	

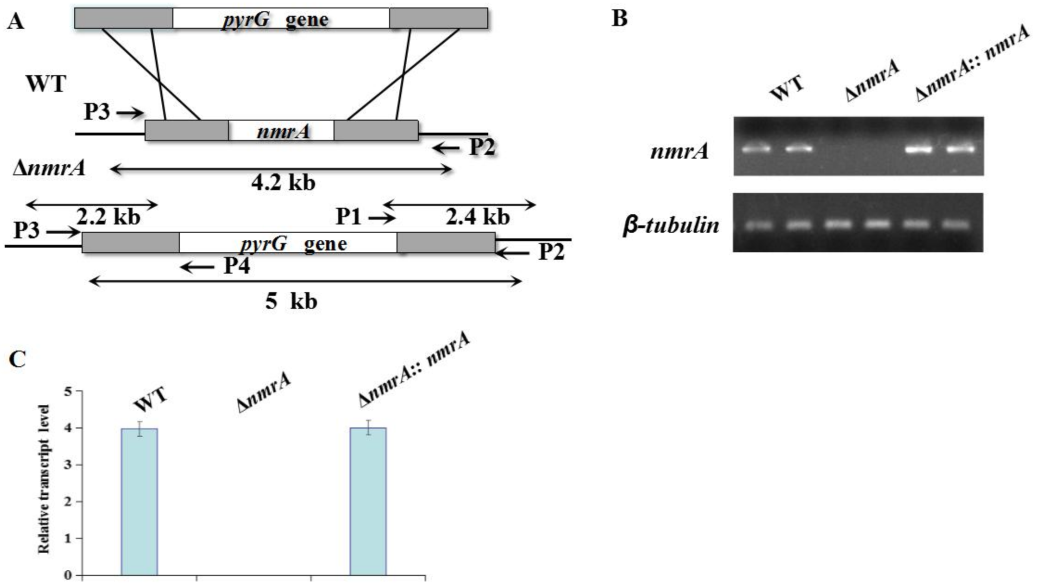

Fig. S1

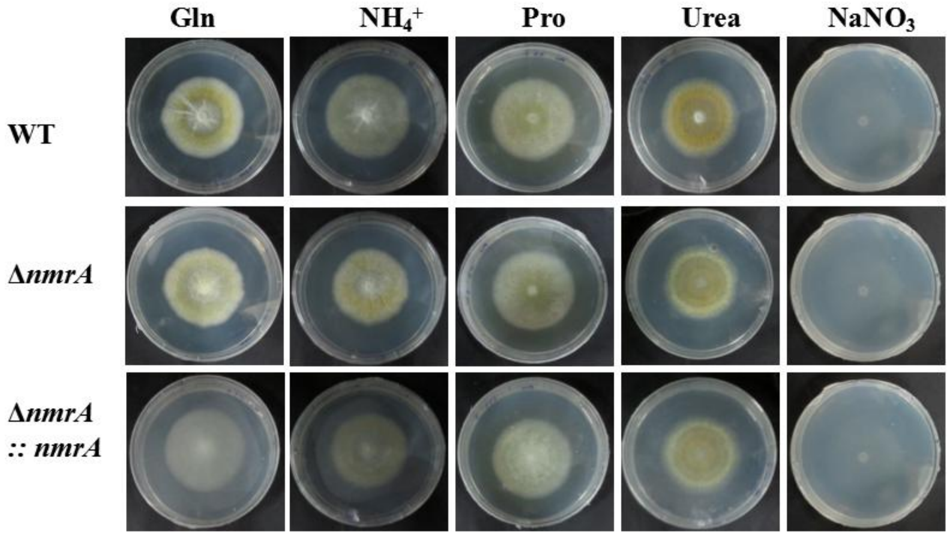

Fig. S2
